# In favour of the definition "adolescents with idiopathic scoliosis": juvenile and adolescent idiopathic scoliosis braced after ten years of age, do not show different end results. SOSORT award winner 2014

**DOI:** 10.1186/1748-7161-9-7

**Published:** 2014-06-27

**Authors:** Sabrina Donzelli, Fabio Zaina, Monia Lusini, Salvatore Minnella, Stefano Negrini

**Affiliations:** 1 ISICO, Italian Scientific Spine Institute, Via Roberto Bellarmino 13/1, 20143 Milan, Italy; 2 Department of Clinical and Experimental Sciences, University of Brescia, Piazzale Spedali Civili, 1, 25123 Brescia, Italy; 3 IRCCS Don Gnocchi ONLUS, via Alfonso Capecelatro, 66 - 20148 Milano, Italy

## Abstract

**Background:**

The most important factor discriminating juvenile (JIS) from adolescent idiopathic scoliosis (AIS) is the risk of deformity progression. Brace treatment can change natural history, even when risk of progression is high. The aim of this study was to compare the end of growth results of JIS subjects, treated after 10 years of age, with final results of AIS.

**Methods:**

Design: prospective observational controlled cohort study nested in a prospective database. Setting: outpatient tertiary referral clinic specialized in conservative treatment of spinal deformities. Inclusion criteria: idiopathic scoliosis; European Risser 0–2; 25 degrees to 45 degrees Cobb; start treatment age: 10 years or more, never treated before. Exclusion criteria: secondary scoliosis, neurological etiology, prior treatment for scoliosis (brace or surgery). Groups: 27 patients met the inclusion criteria for the AJIS, (Juvenile Idiopathic Scoliosis treated in adolescence), demonstrated by an x-ray before 10 year of age, and treatment start after 10 years of age. AIS group included 45 adolescents with a diagnostic x-ray made after the threshold of age 10 years. Results at the end of growth were analysed; the threshold of 5 Cobb degree to define worsened, improved and stabilized curves was considered. Statistics: Mean and SD were used for descriptive statistics of clinical and radiographic changes. Relative Risk of failure (RR), Chi-square and T-test of all data was calculated to find differences among the two groups. 95% Confidence Interval (CI) , and of radiographic changes have been calculated.

**Results:**

We did not find any Cobb angle significant differences among groups at baseline and at the end of treatment. The only difference was in the number of patients progressed above 45 degrees, found in the JIS group. The RR of progression of AJIS was, 1.35 (IC95% 0.57-3.17) versus AIS, and it wasn't statistically significant in the AJIS group, in respect to AIS group (p = 0.5338).

**Conclusion:**

There are no significant differences in the final results of AIS and JIS, treated with total respect of the SRS and SOSORT criteria, in adolescence. Brace efficacy can neutralize the risk of progression.

## Introduction

Idiopathic Scoliosis can be classified according to the age of onset as infantile, juvenile or adolescent [1]. In everyday clinical experience, it is the age of diagnosis, and not of onset, which guide this classification, so if the diagnosis is done before the age of 4, idiopathic scoliosis is defined "infantile", if the diagnosis comes from the age of 4 to the age of 9 years old, then we call "Juvenile" idiopathic scoliosis, and after the age of 10 the idiopathic scoliosis is classified as "adolescent".

The age of scoliosis onset in large extent determines its epidemiology, natural course and response to the treatment [2-5]. The Juvenile form of Idiopathic Scoliosis is generally considered to be more likely to progress and less likely to respond to bracing and more probable to require surgical intervention than the Adolescent form [6]. According to some authors essential differences between the Juvenile and Adolescent Idiopathic Scoliosis include epidemiological data, natural history and response to the treatment. Some years ago Figuereido’s results [3] showed that, due to severe progression 27% to 80% of juvenile curves, required operative treatment, whereas in adolescent type of idiopathic scoliosis risk of progression is much lower and only 0.1% of patients are subjected to operation [2,3,5,7]. The risk of deformity progression can be considered one of the most important factor discriminating juvenile from adolescent type: actually this has great implications in the therapeutical choices and in the definition of therapy goals.

Lonstein and Carlson [8] discovered that in Adolescent Idiopathic scoliosis the curve progression is related to various factors: the pattern and magnitude of the curve, the patient’s age at presentation, the Risser sign, and the patient’s menarchal status. Some years before, Nash [9] demonstrated that the major risk of progression in Juvenile scoliosis is during the prepubertal growth period.

The cause or causes of different age of scoliosis onset and related differences in natural history remain to be elucidated. Recently Nowak [10] hypothesized that some differences between AIS and JIS could be related to mRNA abundance of vitamin D receptor 1 isoform in paravertebral muscles. However their results indicated that there are no difference among the two groups analyzed. The exact meaning of this finding is unknown, but this lack of differences in both analyzed groups suggests precariousness in drawing definite conclusions.

Aside from the risk of progression, there are other factors which have an influence on end of growth curve magnitude. One of the most important factors is surely the treatment. Recently Weinstein [11] declare in conclusion of a multicenter study, that bracing significantly decreased the progression of high risk curves to the threshold for surgery in patients with AIS.

Every day clinical experience and scientific literature contribute to the arising suspicion that the risk of rapid progression of curves is real, but it is not directly correlated to the age of onset, or better the diagnosis age. There are presently very few studies with long term follow up of JIS and even fewer looking specifically at bracing in these subjects.

The main hypothesis considered in this study, is that AIS and JIS treated in adolescence due to the same deformity in Cobb degrees, don’t really differ in term of potential aggressiveness of curves. So the aim of this study was to compare the final results of two groups of scoliosis patients, treated during adolescence, with the same characteristics, the only difference among the two groups are the age of diagnosis of scoliosis, thus defining one group as JIS and the other as AIS.

## Material and method

### Design

This is a retrospective observational study on consecutive outpatients selected from a prospective database started in March 2003. Data were retrieved from a prospective clinical database, all data are collected after subscription of informed consent for the use of data for research purposes. The participation to the present research doesn’t imply any risk or damage for patients, in perfect respect do the Helsinki Declaration. In Italy for this kind of research the ethical committee approval is not required.

### Setting

The setting was an outpatient tertiary referral clinic specialized in conservative treatment of spinal deformities.

### Participants

Out of 1386 idiopathic scoliosis patients, who finished treatment and were recruited in the database before December the 31^st^, of 2013, we found 72 patients, respecting the following inclusion criteria:

• Idiopathic scoliosis

• first evaluation at our Institute

• start and end of therapy (defined as European Risser 3) radiographies available

• no previous brace treatment

• 10 years of age or more

• European Risser sign between 0 and 2

• curve magnitude between 25° and 45°

• girls pre-menarchal or less than 1 year post menarche.

We split patients in two groups according to the age at first diagnosis:

• 45 belonged to the Adolescent Idiopathic Scoliosis Group (AISG)

• 27 assigned to the Juveniles Idiopathic Scoliosis Group (JISG).

JISG included all those patients with a radiographic diagnosis of Juvenile scoliosis never treated before 10 years of age. All these patients were at their first evaluation in our Institute after the age of 10, but they have done x-rays before, thus confirming the diagnosis of Juvenile idiopathic scoliosis.

### Treatments

Most patients involved were treated with one of the rigid braces belonging to the SPORT concept [12], Sibilla and Sforzesco braces, which follow the same biomechanical concept, but differ in the material used for construction. All therapeutical choices were taken according to specific needs following a thorough clinical and radiographical evaluation, made by scoliosis experts, in agreement with the SOSORT guidelines [13]. The therapeutic approach has already been described in previous papers [14,15]; the therapy protocol was a full time prescription of brace wear at the beginning, (18 to 23 hours per day) decided by the specialist according to specific clinical needs of each patients and related to the main goal of the therapy shared by all the team members. Association with physiotherapeutic specific exercises was systematically prescribed to all patients and for the entire duration of treatment, SEAS exercises have to be updated every three months, then a regular exercise performance, at home or followed by a personal trainer, is required with at least two sessions per week (minimum 90 minutes per week). Some patients could decide to follow usual physiotherapy, thus not adhering to SEAS protocol, others decided not to perform any type of exercises. In the data analysis these last two option were classified together, as no SEAS exercises. The follow up visits according to the protocol were scheduled every 6 months.

### Outcomes

We compared clinical and radiographic variations between start and end of therapy. Primary outcome criteria: definition of progressed, stable, improved patients, respecting the threshold of 5 Cobb degrees, in agreement with the SRS criteria for the definition of outcomes. Secondary outcome measures considered: the ATR for the measure of the rib hump and TRACE [16] index for trunk aesthetic evaluation. For TRACE score, it was defined a threshold of three point to define improved, stabilized or progressed subjects, while for ATR the threshold was set at 4 degrees. In a secondary analysis, patients were grouped according to the severity of the major curves, the 30° and 45° Cobb degrees were the chosen thresholds, and the analysis was done respectively at start and at the end of treatment.

### Statistics

Average values and their standard deviation, and percentage were used for descriptive statistics. T-test was used to verify baseline characteristics in the two groups analysed and to check significant differences in Cobb final results. Also Chi-square test has been performed to compare final results in the two groups. Level of significance was set at 0.05.

## Results

In total 72 patients with idiopathic scoliosis have been included in the study, 45 were enrolled in the group of Adolescent idiopathic scoliosis (AISG), and 27 were assigned to the group of Juvenile Idiopathic scoliosis (JISG). Demographical distribution of data, treatment allocation (exercises and brace type) and curves type are summarized in Table 1. No statistically significant difference was found among groups for each parameter.

**Table 1:** **descriptive data of the two samples of patients**, **mean values** (**Standard Deviation**) **and percentages**

	**Mean (SD) %**	**AISG**	**JISG**	**P**
Baseline characteristics	*Age*	12.9 (1.4)	11.4 (1.2)	*NS*
	*Females*	82.2%	88.8%	*NS*
	*Cobb degrees*	32.6 (6.1)	32.7 (6.4)	*NS*
Exercises	*PSSE*: *SEAS*	62.2%	70.4%	*NS*
Brace type	*Sibilla*	37.8%	40.7%	*NS*
	*Sforzesco*	60.0%	59.3%	
	*Others*	2.2%	0.0%	
Curves type	*Single*	11.1%	7.4%	*NS*
	*Th*	11,1%	7,4%	
	*Double*	84.4%	85.2%	
	*Th* + *Lu*	64,4%	74,1%	
	*Th* + *TL*	11,1%	7,4%	
	*Other*	8.9%	3.7%	
	*Triple*	4.4%	7.4%	

No significative differences were found between the two groups analyzed. *SD*: Standard Deviation; P: statistical significance; *NS*: Not Significant. *PSSE*: Physiotherapeutic Scoliosis Specific Exercises. *Th*: Thoracic; *TL*: Thoracolumbar; *Lu*: Lumbar.

**Figure 1:**
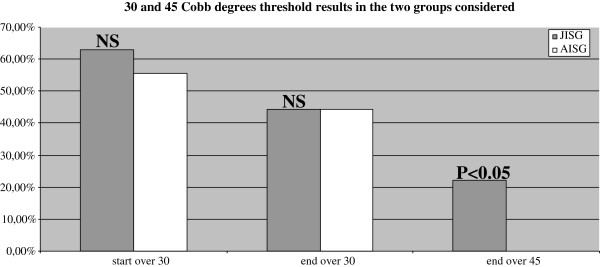
Results according to the 30° and 45° Cobb threshold in AISG and JISG, statistically significant difference was found only for the end over 45° Cobb in JISG.

Final results were analyzed and compared to the starting point. We did not find any statistically significant difference in terms of ATR; TRACE, and Cobb degrees (Table 2).Finally results have been analyzed according to the clinically significant 30 and 45 Cobb degrees thresholds. We did not find statistically significant differences at start and end of treatment for curves above 30° Cobb, (Figure 1) but the number of patients who reached end of treatment above 45° Cobb was significantly higher in JISG.

**Table 2:** **Percentage rate for end point outcomes classified according to the final results as improved**, **stabilized and progressed in both groups**

	**Cobb degrees**			**ATR**			**TRACE**		
	**AISG**	**JISG**	**TOT**	**AISG**	**JISG**	**TOT**	**AISG**	**JISG**	**TOT**
**Improved**	44.4%	29.6%	38.9%	35.6%	55.6%	43.0%	37.8%	33.3%	36.1%
**Stabilized**	48.9%	55.6%	51.4%	64.4%	40.7%	55.5%	60.0%	59.3%	59.7%
**Progressed**	6.6%	14.8%	9.7%	0.0%	3.7%	1.4%	2.2%	7.4%	4.2%
**P value**	**NS**			**NS**			**NS**		

## Discussion

This study has shown in two group of patients totally comparable at start of treatment and in treatment applied, with the only difference of age at diagnosis, that patients with JIS have the same risk of progression as AIS. Also clinical parameters appears similar. The only difference was in the number of patients progressed above 45°, that were all in the JIS group. As a consequence, from this study, JIS does not increase the risk of progression per se, but only that of very important progression.

The classification of scoliosis as juvenile or adolescent only depends on the age at diagnosis. Juvenile idiopathic scoliosis can be considered a transitional form between infantile and adolescent idiopathic scoliosis. Spinal growth is one of the major factors fuelling scoliosis progression, during juvenile period it is fairly steady, while it reaches the peak in prepubertal spurt, corresponding to adolescent period [1].

Literature reported for Juvenile idiopathic scoliosis a worse prognosis and a higher rate of complications during adulthood [6,17].

Although Juvenile scoliosis is considered a challenge for management and evaluation there have been few large scale, long term studies to describe the presentation, problems , and outcomes of different treatments. Few studies reported a wide range of patients requiring surgery, from 27% to 87% [2-4,6,18,19]. Robison and Mac Master reported a progression rate of 97% [2] in a sample of children followed up until skeletal maturity. Figueiredo and James [3,20] confirmed a higher risk of progression in 98 patients reviewed in which 56% required surgical intervention. Some of these authors included in the samples also subjects with associated neurological diseases or intraspinal abnormalities. The presence of secondary forms of scoliosis, can introduce an important bias, actually it is widely accepted that idiopathic scoliosis are less aggressive than secondary ones [19].

The association between Juvenile scoliosis and intraspinal abnormality is still controversial, with some authors recommending for all patients a MR imaging exam to exclude this kind of association and other endorsing the clinical neurological evaluation as reliable to detect these abnormalities [21,22].

Scoliosis treatment is able to change the natural history of scoliosis [11], so in the analyses of risks of treated patients brace efficacy and patients management can play an important role.

All these issues contribute to the interpretation of the current results. This research aimed to find differences in final results between a group of adolescents with idiopathic scoliosis, and a group of adolescents with a juvenile scoliosis diagnosis, starting from the hypothesis that due to the supposed major risks of progression, the delay in the intervention would have rise worst final results. Actual results disproved the main hypothesis, as no difference was found among the AISG and JISG.

A main point to be noted to compare this study to the others in the literature is the low rate of high degree curves (considered surgical) in this sample (22% in JISG when compare to 27-97% in other studies) [2-4,6,11,18,19]. Obviously the retrospective design could influence this result, even if other studies were retrospective as well [2,3,6,17]; another possible explanation is the good results already reported by the authors [14,23] in comparison to many others [24-28], but not all [29,30]. Possibly these good results are due to the team approach [31], exercises usage [32], bracing compliance [33] and perhaps also efficacy [34], and patients’ management [34]. Therefore in the studied sample it is possible that the good treatment is the most important factor affecting results. It is possible that a high quality management of scoliosis is able to compensate the risks correlated to the delayed intervention.

Another possible explanation is that other studies concentrated mainly on worst progressions, and not only on progression in general. Again, a good therapeutic approach can limit these worst progressions, so reducing the differences between the two groups.

In summary, Juvenile idiopathic scoliosis can have a very similar presentation to the adolescent form, with similar final results. All these similarities highlight the limitations of a classification based on the age of diagnosis, which can hardly admit prognosis estimation. So the present study endorse the definition "adolescent with idiopathic scoliosis", which implies the age when treatment is started, and seems more feasible and realistic than "adolescent idiopathic scoliosis", which comes from the age at diagnosis.

Another interesting classification of scoliosis, with a better adherence to real clinical everyday activity, is the one proposed by Dickson and Archer who believed that true juvenile scoliosis was rare enough not to warrant a separate category. They suggested to classify scoliosis as early onset, for that forms presenting before the age of 5 years and late onset for all the other scoliosis presenting after the age of 5 [35,36].

The main limitation of the present study is the small size of the two groups, in particular the JISG was quite small. This can allows some bias correlated to a type two error. On the other hand the small sample size of the JISG is justified by the infrequence of cases of juvenile idiopathic scoliosis remaining untreated until the adolescent age. Actually, it is the first time that such a comparison was done, and this primacy is one of the major strength of the present study.

Despite the awareness that results must be interpreted with caution, some very interesting insights are offered; further studies with larger samples and long term results are needed to better understand the important factors which contribute to Juvenile Idiopathic Scoliosis progression, regardless to the age of diagnosis.

## Conclusion

The present study showed that in JIS there are some of the worst cases (worst Cobb degrees final results), but also that the time of diagnosis can not be considered a predictor of progression of idiopathic scoliosis. Scoliosis is a complex disease and various factors are involved in the risk of progression of deformity and to limit this complexity to the age of diagnosis is surely hazardous. The efficacy of bracing, the adherence to the treatment, the correct therapeutical choices as the management of the patients and family involved into the treatment team can contribute to stop or decrease the progression of scoliosis independently from the age of diagnosis.

## Competing interests

The authors declared that they have no competing interests.

## Authors’ contributions

SD developed the research project, analyzed literature current knowledge, and manage the retrieval of data. Then lead the statistical analysis, and interpreted results and made the conclusion of the study. FZ contributed in the development of the research question, in the study design and in data analysis, the statistical analysis and interpretation of results. ML helped in data retrieval and literature review. SM contributed in recovering data. SN, developed the main research question, planned the design of the study and helped in the interpretation of results, discussion and conclusion. He reviewed the final version of the paper. All authors read and approved the final manuscript.
